# HDAC7 promotes cardiomyocyte proliferation by suppressing myocyte enhancer factor 2

**DOI:** 10.1093/jmcb/mjae044

**Published:** 2024-10-11

**Authors:** Jihyun Jang, Mette Bentsen, Jin Bu, Ling Chen, Alexandre Rosa Campos, Mario Looso, Deqiang Li

**Affiliations:** Center for Cardiovascular Research, Abigail Wexner Research Institute, Nationwide Children's Hospital, Columbus, OH 43205, USA; Department of Pediatrics, The Ohio State University College of Medicine, Columbus, OH 43205, USA; Bioinformatics Core Unit (BCU), Max Planck Institute for Heart and Lung Research, 61231 Bad Nauheim, Germany; Center for Cardiovascular Research, Abigail Wexner Research Institute, Nationwide Children's Hospital, Columbus, OH 43205, USA; Department of Physiology, University of Maryland School of Medicine, Baltimore, MD 21201, USA; Proteomics Facility, Sanford Burnham Prebys Medical Discovery Institute, La Jolla, CA 92037, USA; Bioinformatics Core Unit (BCU), Max Planck Institute for Heart and Lung Research, 61231 Bad Nauheim, Germany; Center for Cardiovascular Research, Abigail Wexner Research Institute, Nationwide Children's Hospital, Columbus, OH 43205, USA; Department of Pediatrics, The Ohio State University College of Medicine, Columbus, OH 43205, USA

**Keywords:** HDAC7, dedifferentiation, proliferation, cardiomyocyte

## Abstract

Postnatal mammalian cardiomyocytes (CMs) rapidly lose proliferative capacity and exit the cell cycle to undergo further differentiation and maturation. Cell cycle activation has been a major strategy to stimulate postnatal CM proliferation, albeit achieving modest effects. One impediment is that postnatal CMs may need to undergo dedifferentiation before proliferation, if not simultaneously. Here, we report that overexpression of *Hdac7* in neonatal mouse CMs results in significant CM dedifferentiation and proliferation. Mechanistically, we show that histone deacetylase 7 (HDAC7)-mediated CM proliferation is contingent on dedifferentiation, which is accomplished by suppressing myocyte enhance factor 2 (MEF2). *Hdac7* overexpression in CM shifts the chromatin state from binding with MEF2, which favors the transcriptional program toward differentiation, to binding with AP-1, which favors the transcriptional program toward proliferation. Furthermore, we found that HDAC7 interacts with minichromosome maintenance complex components to initiate cell cycle progression. Our findings reveal that HDAC7 promotes CM proliferation by its dual action on CM dedifferentiation and proliferation, uncovering a potential new strategy for heart regeneration/repair.

## Introduction

Significant loss of cardiomyocytes (CMs) that occurs during the pathogenesis and progression of many forms of cardiovascular disease can eventually lead to heart failure. This is at least partially attributable to the limited regenerative capacity of adult CMs in mammals. New CMs can be derived through proliferation of endogenous pre-existing CMs under both physiological and pathological conditions, although the rate is very low (0.5%–2% estimated annual turnover) (Eschenhagen et al., 2017). The key impediment is that adult CMs are terminally differentiated and have exited the cell cycle. Various strategies have been attempted to reactivate the cell cycle to enhance endogenous CM proliferative capacity including manipulating signaling pathways (Xin et al., 2013; Morikawa et al., 2017; Nakada et al., 2017; Nguyen et al., 2020), cell cycle regulators (Mohamed et al., 2018), microRNAs (Tian et al., 2015), and extracellular matrix proteins (Bassat et al., 2017). While the results are encouraging, there remains a challenge to achieve significant new myocardium without elevated polyploidy or multinucleation. Dedifferentiation is also increasingly recognized as a prerequisite for CM proliferation (Jopling et al., 2010; Wang et al., 2017). Adult zebrafish CMs fail to proliferate upon injury if dedifferentiation is blocked (Jopling et al., 2010). Recent mouse and human heart studies suggest that CMs adjacent to the injury site possess higher regenerative capacity, demonstrating characteristics of dedifferentiation and a distinctive transcriptional profile, compared to distal CMs (Lin et al., 2014; Wang et al., 2017; van Duijvenboden et al., 2019). Cell dedifferentiation is a process by which cells undergo reverse transformation from a partially or terminally differentiated stage to a less differentiated stage within their lineage. The dedifferentiated state affords a high degree of plasticity, enabling cells to undergo drastic events such as proliferation (Ouladan and Gregorieff, 2021). Resetting adult CMs to a dedifferentiated state (e.g. dissociation of sarcomeres and adhesion junctions; downregulation of cardiac sarcomeric genes) has been suggested to be tightly associated with cardiac regenerative capacity (Bongiovanni et al., 2021; Zhu et al., 2021). Analogous to CM differentiation, regulation of CM dedifferentiation is complex and subject to modulation of multiple cardiac transcriptional masters such as myocyte enhance factor 2 (MEF2) family members and their targets (Wang et al., 2017; Padula et al., 2021). It is enticing to speculate whether the regenerative potential in postnatal mammalian CMs can be activated when they are reset to dedifferentiation and cell cycle activation. However, the journey to achieve cell dedifferentiation and cell cycle activation in postnatal CMs appears daunting.

Chromatin state is associated with many physiological and pathological processes (e.g. organ development, aging, and cancer) (Paige et al., 2012; Wamstad et al., 2012; Sen et al., 2016; Liu et al., 2019; Prieto and Maeshima, 2019), is highly dynamic, and subject to remodeling by a variety of chromatin remodeling factors including histone posttranslational modifiers (Prieto and Maeshima, 2019). Chromatin structure and state often dictate cell fate and behavior (Cruciani et al., 2019; Subramaniam et al., 2019; Yi et al., 2019). The transition made during CM development and proliferation is largely dependent on high-order regulation of chromatin status through histone posttranslational modifications, including acetylation/deacetylation, that simultaneously regulate numerous cardiac sarcomeric and cell cycle genes (Paige et al., 2012; Wamstad et al., 2012). Loss of chromatin accessibility may underlie the failure of regenerative program reactivation in adult CMs (Quaife-Ryan et al., 2017). Another recent comprehensive study demonstrated the significant alteration of chromatin states during various stages of adult zebrafish heart regeneration (Cordero et al., 2024). Collectively, as these findings suggest that epigenetic changes may underlie adult CM cell cycle exit (Quaife-Ryan et al., 2016), manipulating chromatin remodeling factors may stimulate postnatal CM proliferation.

Histone deacetylase 7 (HDAC7) belongs to the class IIa HDAC family that consists of four members: HDAC4, HDAC5, HDAC7, and HDAC9 (Verdin et al., 2003). Unlike the ubiquitous class I HDACs, class IIa HDACs have tissue- and organ-specific expression patterns (Di Giorgio and Brancolini, 2016). All class IIa HDACs contain a highly conserved catalytic domain that primarily mediates the recruitment of multiprotein complexes in lieu of deacetylase activity (Shen et al., 2023). Class IIa HDACs contain an MEF2-binding domain allowing the interaction with MEF2 and suppression of its transcriptional activity, which suggests that they have the capacity to affect CM differentiation (Verdin et al., 2003; Clocchiatti et al., 2013; Parra, 2015). Unlike other class IIa HDACs that are expressed at all stages of CMs, HDAC7 is expressed in embryonic CMs but not in adult CMs, suggesting that it may have a unique function in regulating CM proliferation and/or differentiation (Zhang et al., 2002; Chang et al., 2004, 2006; Wright and Menick, 2016; Helmstadter et al., 2021; Han  et al., 2022; Liu et al., 2022). HDAC7 has been shown to be crucial for maintaining vascular integrity during heart development (Chang et al., 2006), and regulating myoblast differentiation by interacting with MEF2 to repress skeletal muscle gene expression (Martin et al., 2009; Gao et al., 2010). Upregulation of HDAC7 has been shown to promote epithelial and tumor cell proliferation, although the underlying mechanisms are unclear (Zhu et al., 2011; Clocchiatti et al., 2015; Cutano et al., 2019; Sang et al., 2019). HDAC7 can function either as a transcriptional repressor or activator in a tissue-dependent manner (Chang et al., 2006; Gao et al., 2010; Azagra et al., 2016; Cutano et al., 2019; Sang et al., 2019).

In the present study, we investigated the potential role of HDAC7 in stimulating postnatal CM dedifferentiation and proliferation. Our findings offer key insight into a novel therapeutic strategy for promoting heart regeneration.

## Results

### HDAC7 promotes CM proliferation

First, we determined the expression profile of class IIa HDACs. HDAC4, HDAC5, HDAC7, and HDAC9 were all expressed in E10.5 embryonic CMs, albeit with difference in expression localization: HDAC4 and HDAC5 were predominantly localized in the cytoplasm, while HDAC7 and HDAC9 were predominantly in the nucleus (Supplementary Figure S1A). Interestingly, HDAC4, HDAC5, and HDAC9 expression was maintained in adult CMs, whereas HDAC7 expression was absent (Supplementary Figure S1B). Our findings are consistent with previous reports (Zhang et al., 2002; Chang et al., 2006; He et al., 2020; Helmstadter et al., 2021). This finding also suggests that HDAC7 may be associated with the loss of proliferative capacity in adult CMs.

**Figure 1:**
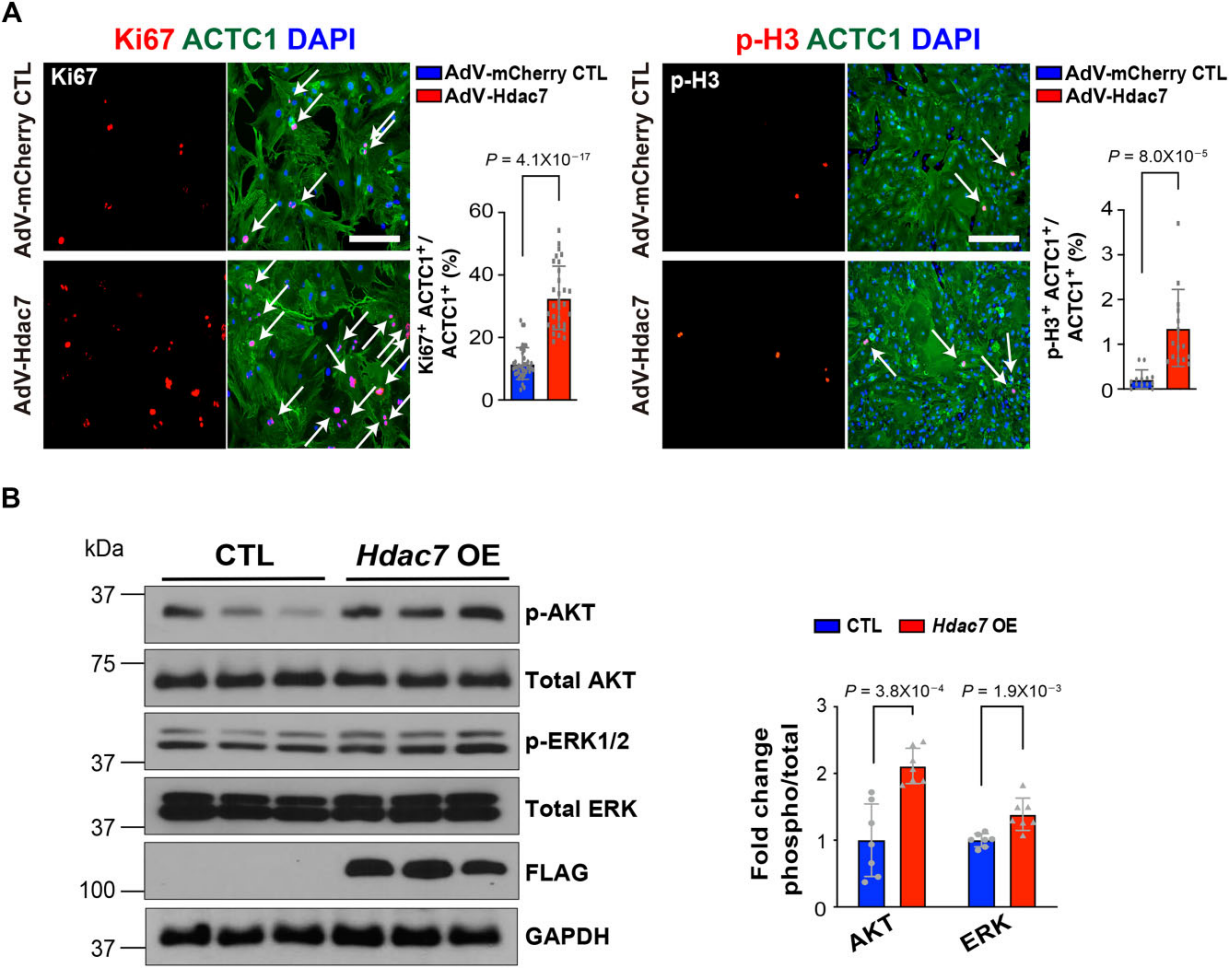
HDAC7 promotes CM proliferation. (**A**) HDAC7 promotes proliferation of NMCMs. Cultured NMCMs were transfected with adenoviruses (working titer: 1 × 10^7^ GC/ml) harboring mCherry (CTL) or *Hdac7-*mCherry (*Hdac7* OE). By immunofluorescence staining, NMCMs were identified by ACTC1^+^, and percentages of Ki67^+^ and p-H3^+^ NMCMs were quantified. Independent samples: % of Ki67^+^, *n* = 39 for CTL and *n* = 30 for *Hdac7* OE; % of p-H3^+^, *n* = 13 for CTL and *n* = 15 for *Hdac7* OE. *P*-values were determined by unpaired Student's *t*-test. Scale bar, 125 μm (for Ki67) or 275 μm (for p-H3). (**B**) Activation of p-ERK and p-AKT by *Hdac7* OE. Representative western blot images and the densitometric quantification of p-ERK and p-AKT are shown. *n* = 7 in each group. *P*-values were determined by unpaired Student's *t*-test.

We hypothesized that HDAC7 regulates the switch between CM proliferation and differentiation. As neonatal mouse CMs (NMCMs) are transitioning from being highly proliferative and immature to being less proliferative and more mature (Porrello and Olson, 2014), we used them as the main platform to test our hypothesis. To determine whether *Hdac7* overexpression (OE) can induce CM proliferation, we treated cultured NMCMs with adenoviral *Hdac7*-mCherry or adenoviral mCherry (control, CTL) and evaluated cell cycle activation 72 h after treatment. Expression of *Hdac7*-mCherry and mCherry is driven by the *Tnnt2* promoter to restrict expression to CMs. We found significantly higher percentages of p-H3^+^ CMs and Ki67^+^ CMs (Figure 1A) and upregulated cell growth signaling (p-ERK and p-AKT) (Figure 1B) in *Hdac7* OE NMCMs compared to CTL NMCMs. One week after transfection, *Hdac7* OE NMCMs appeared phenotypically normal as compared to CTL NMCMs, i.e. beating was regular, sarcomere structure (assessed by TNNT2 immunostaining) appeared intact, and there was no excessive cell death (assessed by terminal deoxynucleotidyl transferase dUTP nick end labeling) (Supplementary Figure S2), suggesting that transient *Hdac7* OE does not compromise CM structure, function, or viability. To determine whether HDAC7 deacetylase activity is required to regulate CM proliferation, we treated NMCMs with TMP269, an HDAC7-specific inhibitor (Lobera et al., 2013). TMP269 treatment did not attenuate NMCM proliferation determined by the percentage of Ki67^+^ NMCMs (Supplementary Figure S3), suggesting that the proliferation-promoting activity of HDAC7 is independent of its deacetylase activity. To determine whether HDAC7 is required for normal CM proliferation, we efficiently knocked down *Hdac7* with shRNA and found that the percentage of Ki67^+^ CMs was significantly lower in *Hdac7* knockdown NMCMs compared to CTL NMCMs (Supplementary Figure S4A). Western blot analysis showed a significant reduction in the protein levels of cell cycle activators (e.g. c-MYC and PCNA) (Supplementary Figure S4B), confirming the decreased proliferative capacity in *Hdac7* knockdown NMCMs.

### Hdac7 OE induces cell cycle activation and CM dedifferentiation

**Figure 2:**
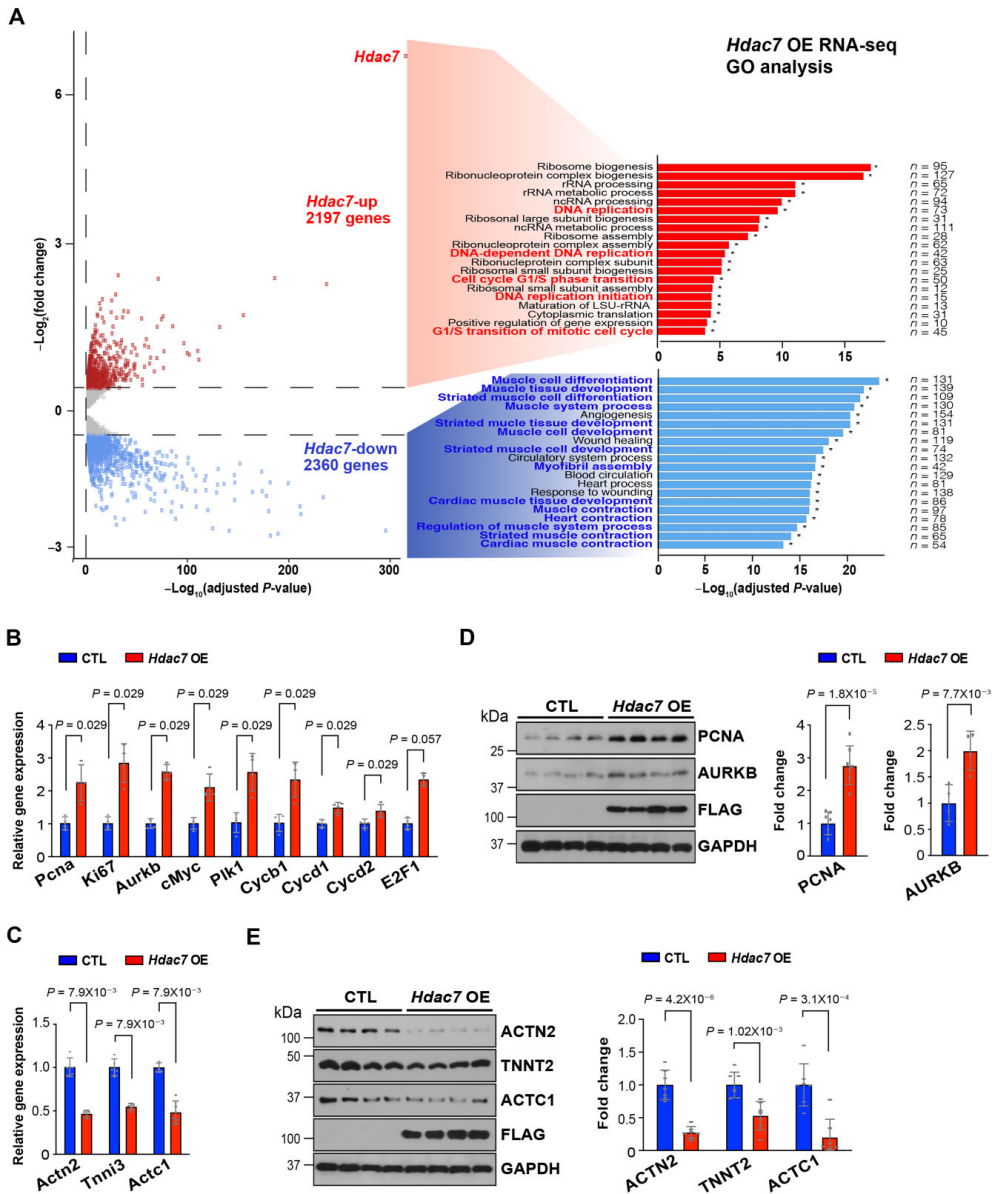
HDAC7 downregulates cardiac sarcomeric genes and upregulates cell cycle genes. Cultured NMCMs were transfected with *Hdac7-*mCherry (*Hdac7* OE) or mCherry (CTL) adenoviruses for 24 h (working titer: 1 × 10^7^ GC/ml). (**A**) Volcano plot and GO pathway analyses following RNA-seq on *Hdac7* OE and CTL NMCMs. *n* = 4 in each group. Fold changes were calculated by RPKM (reads per kb of transcript per million mapped reads) per gene in the *Hdac7* OE group divided by the mean RPKM per gene in the CTL group. Significantly downregulated genes are shown in light blue, and significantly upregulated genes are shown in red. Cut-off criteria: adjusted *P*-value <0.01. (**B** and **C**) Quantification of mRNA expression of cell cycle genes (**B**, *n* = 4 in each group) and cardiac sarcomeric genes (**C**, *n* = 5 in each group) by qRT-PCR. *Gapdh* was used as cDNA loading control. (**D** and **E**) Quantification of PCNA (*n* = 7 in each group), AURKB (*n* = 4 in each group), and ACTN2, TNNT2, and ACTC1 (*n* = 7 in each group) by western blotting. GAPDH was used as protein loading control. *P*-values were determined by the Mann–Whitney *U* test (**B**–**D**) or unpaired Student's *t*-test (**E**).

We next sought to understand the underlying molecular mechanisms by which HDAC7 induces CM proliferation. Bulk RNA sequencing (RNA-seq) and Gene Ontology (GO) analyses on CTL and *Hdac7* OE NMCMs identified 2360 downregulated genes and 2197 upregulated genes (Figure 2A). Interestingly, GO pathway analyses identified that genes involved in cell cycle activation and cell proliferation (e.g. *Aurkb, Pcna*, and *Ki67*) were significantly upregulated, while genes involved in CM differentiation (e.g. *Actn2, Tnnt2*, and *Actc1*) were significantly downregulated (Figure 2A). We validated these findings by quantitative real-time polymerase chain reaction (qRT-PCR) (Figure 2B and C) and western blotting (Figure 2D and E). These results suggest that HDAC7 induces both cell cycle activation and CM dedifferentiation.

### Interaction with MEF2 is required for CM dedifferentiation and proliferation by HDAC7

**Figure 3:**
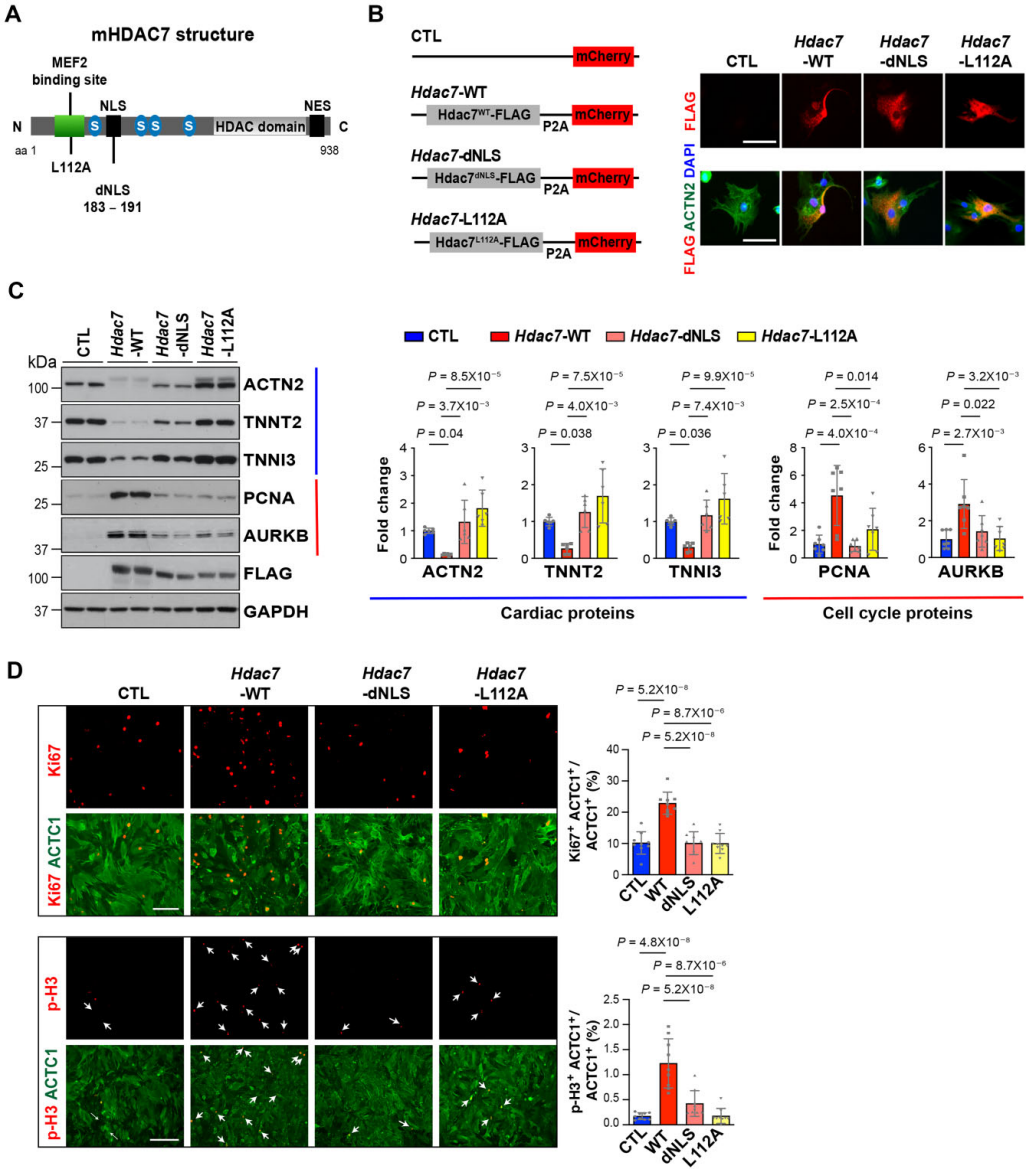
Binding to MEF2 is required for HDAC7 to promote CM proliferation. (**A**) Schematic illustration of mouse HDAC7 protein. (**B**) Expression of various *Hdac7* adenoviral constructs in NMCMs shown by immunofluorescence. Scale bar, 60 μm. (**C**) Expression of cardiac proteins (ACTN2, TNNT2, and TNNI3; *n* = 6 in each group) and cell cycle proteins (PCNA and AURKB; *n* = 7 in each group) in NMCMs 3 days post-transfection. GAPDH was used as protein loading control. *P*-values were determined by one-way ANOVA followed by the Tukey *post hoc* test. (**D**) Proliferation of NMCMs transfected with various *Hdac7* constructs was evaluated by immunofluorescence staining. Independent samples: % of Ki67^+^, *n* = 9 in each group; % of p-H3^+^, *n* = 9 in each group. *P*-values were determined by one-way ANOVA followed by the Tukey *post hoc* test. Scale bar, 125 μm (for Ki67) or 275 μm (for p-H3).

HDAC7 induces skeletal muscle dedifferentiation through its interaction with MEF2 in the nucleus (Gao et al., 2010). To determine whether the interaction with MEF2 is critical for HDAC7 to stimulate CM proliferation, we transfected NMCMs with *Hdac7*-dNLS adenovirus (*Hdac7* with deletion of nuclear localization sequence) and *Hdac7*-L112A adenovirus (*Hdac7* with MEF2-binding site mutation: leucine replaced by alanine at amino acid 112) (Figure 3A). We confirmed that *Hdac7*-dNLS was retained in the cytoplasm and that neither *Hdac7*-dNLS nor *Hdac7*-L112A suppressed the expression of cardiac sarcomeric proteins including ACTN2, TNNT2, and TNNI3 (Figure 3B and C). We also found that the percentage of Ki67^+^ or p-H3^+^ NMCMs was significantly lower in the NMCMs transfected with *Hdac7*-dNLS or *Hdac7*-L112A adenovirus compared to the NMCMs transfected with *Hdac7*-WT adenovirus (Figure 3D). These findings are consistent with decreased expression of cell cycle activators (e.g. PCNA and AURKB) in *Hdac7*-dNLS or *Hdac7*-L112A-treated NMCMs compared to *Hdac7*-WT-treated NMCMs (Figure 3C). These results suggest that CM dedifferentiation controlled by nuclear interaction between HDAC7 and MEF2 is critical for HDAC7 to drive CM proliferation.

### HDAC7 functions as a molecular switch of TF occupancy from MEF2 to AP-1

**Figure 4:**
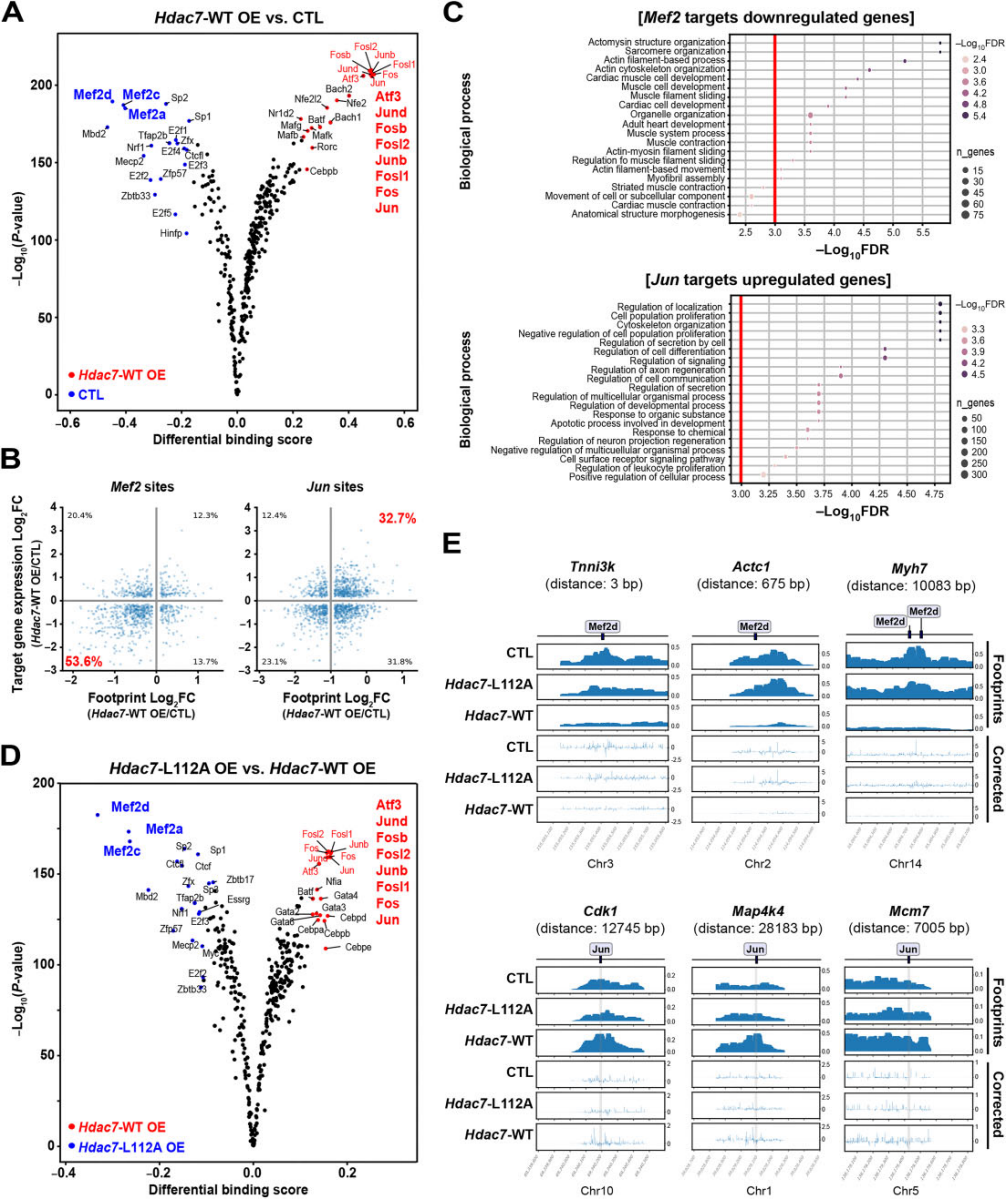
HDAC7 functions as a molecular switch of TF occupancy from MEF2 to AP-1. (**A**) *Hdac7* OE causes TF occupancy prediction shift. ATAC-seq was performed on NMCMs treated with *Hdac7*-WT or CTL adenoviruses. TOBIAS was used for differential binding score analysis of the ATAC-seq dataset. Volcano plot shows the differential TF occupancy prediction, with blue and red points depicting motifs that have enriched TF footprints CTL and in *Hdac7*-WT OE NMCM genomes, respectively. (**B**) Integration analysis of ATAC-seq and RNA-seq datasets. Scatterplots show the gene expression changes near MEF2- and Jun-binding sites found in gene promoters. Footprint Log_2_FC and RNA Log_2_FC (expression) represent the matched changes between the *Hdac7*-WT OE group and the CTL group for footprints and gene expression, respectively. (**C**) GO analysis of downregulated MEF2-binding site targets (*n* = 844) and upregulated Jun-binding site targets (*n* = 2053). (**D**) Volcano plot shows the differential TF occupancy prediction between *Hdac7*-WT and *Hdac7*-L112A by TOBIAS analysis, with blue and red points depicting motifs that have enriched TF footprints in *Hdac7*-L112A OE and *Hdac7*-WT OE NMCM genomes, respectively. Data were derived from two independent samples for each group. (**E**) Genomic tracks indicate the binding sites at the *Mef2d* or *Jun* locus in CTL, *Hdac7*-WT OE, and *Hdac7*-L112A OE NMCMs. Solid black boxes show target gene promoters and respective tracks for TOBIAS footprint scores or corrected cutsite signals.

Reestablishment of chromatin accessibility is necessary for cardiac regeneration (Quaife-Ryan et al., 2017). To determine whether HDAC7 dictates chromatin landscape, thus regulating CM chromatin accessibility to transcription factors (TFs), we performed assay for transposase-accessible chromatin coupled to high-throughput sequencing (ATAC-seq) on *Hdac7* OE NMCMs. As expected, accessible chromatin regions were primarily located in promoters, gene bodies, and intergenic regions (Supplementary Figure S5A). To predict TF occupancy in our *Hdac7* OE NMCM ATAC-seq dataset, initial homer motif enrichment analysis indicated that the occupancy of MEF2 TF family motifs was replaced by those from multiple members of the AP-1 TF family members (e.g. *Atf3* and *JunB*) (Supplementary Figure S5B). Through transcription factor occupancy prediction by investigation of ATAC-seq signal (TOBIAS), a more comprehensive computational framework for footprinting analysis (Bentsen et al., 2020), we confirmed the significant reduction of putative MEF2-binding motifs in accessible chromatin regions in *Hdac7* OE NMCMs. Concurrently, AP-1 motifs with a high putative TF-binding score became more accessible in *Hdac7* OE NMCMs (e.g. *Atf3, JunB*, and *Fos*) (Figure 4A). These findings suggest that HDAC7 switches CM gene transcriptional programs from MEF2-driven to AP-1-driven.

To determine whether selective open chromatin regions identified by ATAC-seq analysis correlated with CM-specific gene expression, we integrated our ATAC-seq data with RNA-seq data. This comparison showed that the majority of genes associated with decreased MEF2 binding are downregulated in *Hdac7*-WT OE NMCMs compared with CTL NMCMs, whereas genes with increased Jun binding are correspondingly upregulated (Figure 4B). As expected, GO pathway analyses identified that the downregulated MEF2-related pathways are involved in muscle development, whereas the upregulated AP-1 (Jun) targets are associated with cell proliferation (Figure 4C). These results imply that HDAC7 switches the gene program from MEF2-driven differentiation to AP-1-driven cell proliferation.

Next, we sought to determine whether the HDAC7 interaction with MEF2 accounts for the shifted chromatin accessibility by performing a comparative analysis of ATAC-seq datasets between *Hdac7*-WT and *Hdac7*-L112A OE NMCMs (Figure 4D). Unlike *Hdac7*-WT, we found that *Hdac7*-L112A OE did not affect MEF2 binding to the motifs in several sarcomere genes (*Myh7, Actc1*, and *Tnni3k*) (Figure 4E). Additionally, there was no increased AP-1 chromatin accessibility to known targets including *Cdk1, Map4k4*, and *Mcm7* (Figure 4E). These results are consistent with the above findings from cell differentiation and proliferation assessment (Figure 3), corroborating the critical roles of CM dedifferentiation and proliferation promoted by HDAC7.

### HDAC7 interacts with MCM7 and promotes CM proliferation

**Figure 5:**
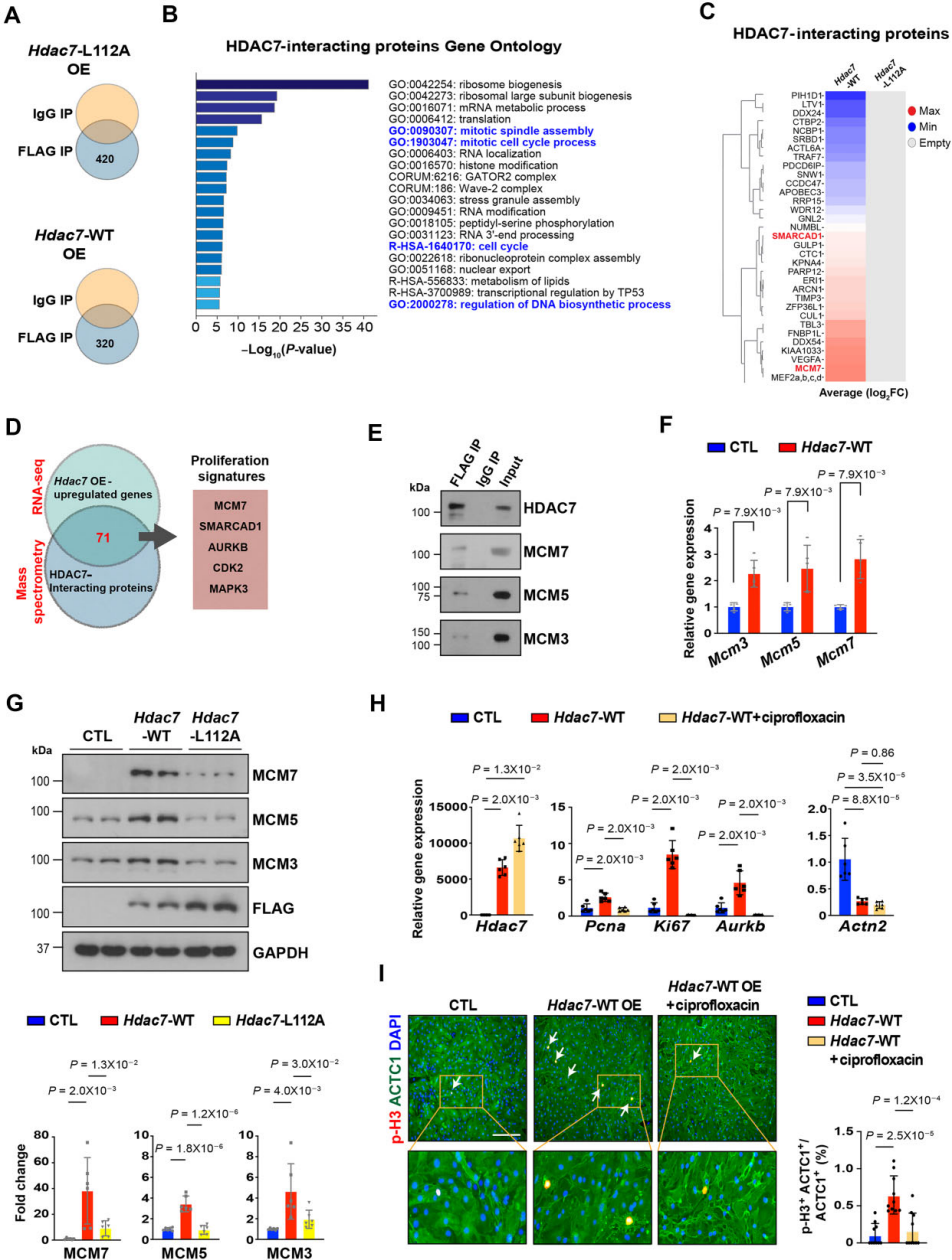
HDAC7 interacts with and upregulates MCM7. (**A**) Schematic of the experimental approach to identify HDAC7-interacting proteins in *Hdac7*-WT or *Hdac7*-L112A OE NMCMs by affinity purification followed by MS. (**B**) GO analysis of HDAC7-interacting proteins. The brightness of each color corresponds to the magnitude of the difference when compared with the overall average value (Log_2_FC). (**C**) The heatmap of differential HDAC7-interacting proteins between *Hdac7*-WT and *Hdac7*-L112A OE NMCMs. (**D**) Intersection of *Hdac7* OE-upregulated genes in RNA-seq dataset and HDAC7-interacting proteins in MS dataset. Pie chart depicts the overlapping signatures including MCM7 and SMARCAD1. (**E**) Co-IP assay of HDAC7 with MCM3, MCM5, and MCM7 in *Hdac7* OE NMCMs. (**F**) Quantification of gene expression of *Mcm3, Mcm5*, and *Mcm7* in *Hdac7* OE NMCMs by qRT-PCR. *Gapdh* was used as cDNA loading control. *n* = 5 for each group. *P*-values were determined by the Mann–Whitney *U* test. (**G**) Quantification of MCM3, MCM5, and MCM7 in *Hdac7-*WT and *Hdac7*-L112A OE NMCMs by western blotting. GAPDH used as protein loading control. *n* = 6 for each group. *P*-values were determined by unpaired Student's *t*-test. (**H** and **I**) *Hdac7*-WT OE NMCMs were cultured with or without ciprofloxacin (Sigma, #1134313; working concentration: 5 μM; treatment duration: 48 h). (**H**) Quantification of cell cycle gene expression by qRT-PCR. *Gapdh* was used as cDNA loading control. *n* = 6 for each group. *P*-values were determined by unpaired Student's *t*-test. (**I**) Proliferation was assessed by immunofluorescence staining and the percentage of p-H3^+^ NMCMs was quantified. *n* = 10 in each group. *P*-values were determined by one-way ANOVA followed by the Tukey *post hoc* test. Scale bar, 275 μm.

HDAC7 lacks intrinsic DNA-binding capacity and is recruited to target genes via complexing with transcriptional activators and repressors. To identify additional HDAC7-interacting proteins that may contribute to HDAC7 capacity to promote cell proliferation and/or dedifferentiation, we immunoprecipitated HDAC7 and performed liquid chromatography–mass spectrometry (LC–MS) on lysates from *Hdac7*-WT and *Hdac7-*L112A OE NMCMs (Figure 5A). GO analysis of the *Hdac7*-WT OE protein interactome revealed that HDAC7-interacting proteins are involved in cell cycle activation and cell proliferation (Figure 5B). Interestingly, *Hdac7*-WT OE but not *Hdac7*-L112A OE NMCMs showed strong interaction between HDAC7 and cell proliferation initiator complexes including minichromosome maintenance protein 7 (MCM7) and SWI/SNF-related, matrix-associated actin-dependent regulator of chromatin, subfamily A, containing DEAD/H box 1 (SMARCAD1) (Figure 5C). Interestingly, many hits, including the top hit, MCM7, along with a few other cell cycle activators (SMARCAD1, AURKB, CDK2, and MAPK3), were also significantly upregulated in *Hdac7* OE NMCMs (Figure 5D). MCM7, a replicative helicase, plays an important role in DNA replication by forming MCM complexes with other members of the MCM2-7 protein family (Ishimi, 2018). As expected, other MCM complexes, such as MCM3 and MCM5, also interacted with *Hdac7* as shown by their co-immunoprecipitation (co-IP) (Figure 5E). Expression of these MCM proteins was increased in *Hdac7*-WT OE NMCMs, whereas *Hdac7*-L112A OE abolished the upregulation of these MCM proteins (Figure 5F and G). In addition, *Hdac7*-L112A did not interact with MCM7 (Supplementary Figure S6). To further probe whether the interaction with MCM complexes is critical for HDAC7 to activate the cell cycle, we applied ciprofloxacin (Simon et al., 2013) to block the DNA helicase activity of the MCM2-7 complex in *Hdac7* OE NMCMs. Ciprofloxacin treatment significantly blunted the cell proliferation-promoting effect by HDAC7, determined by the percentage of p-H3^+^ NMCMs (Figure 5H and I), suggesting that HDAC7 induces cell proliferation through its interaction with MCM complexes.

## Discussion

In this report, we demonstrated that *Hdac7* OE elicits CM proliferation. HDAC7 achieved this effect by modifying chromatin accessibility and gene programs. We also discovered that HDAC7 interaction with MEF2 is critical to reset CM to the dedifferentiated state, which is required to induce CM proliferation. Unexpectedly, we found that HDAC7 interacted with MCM complexes to activate the cell cycle from a very early step (unwinding double-stranded DNA). By employing this dual mechanism, HDAC7 promotes CM proliferation.

Although dedifferentiation is a regressive process in which cells regain primitive phenotypes (Yao and Wang, 2020), fully mature adult CMs need to undergo dedifferentiation before reentering the cell cycle. It is quite intriguing that HDAC7 lost its proliferation-promoting capacity when dedifferentiation was blocked (Figure 3). Our results are consistent with early findings in zebrafish where perturbation of CM dedifferentiation blocks heart regeneration (Jopling et al., 2010). In fact, CM undergoing dedifferentiation is occasionally mentioned in murine heart regeneration studies that manipulated Hippo–Yap pathway (von Gise et al., 2012; Monroe et al., 2019), yet without deeper exploration from a mechanistic perspective. A more recent study also observed CM undergoing dedifferentiation while stimulating proliferation with a small molecule cocktail (Du et al., 2022). Altogether, these data suggest that dedifferentiation is a prerequisite for CMs to undergo proliferation. How to induce dedifferentiation warrants important strategic consideration when developing new methods to promote CM proliferation (not just cell cycle activation). Managing the degree and duration of CM dedifferentiation is also critical, as excessive dedifferentiation could compromise CM contractility, thus compromising cardiac function.

We found that HDAC7 orchestrates a unique set of gene transcriptional programs. TF footprint analysis from ATAC-seq data showed that patterns of TF-binding motifs in *Hdac7* OE CMs are switched from MEF2 to AP-1 (Figure 4), which aligned well with transcriptional changes of the respective target genes revealed by RNA-seq (Figure 2). AP-1 can be transiently and rapidly activated in response to a wide variety of cellular stimuli. AP-1 opens chromatin accessibility for genes that promote CM proliferation during heart regeneration in zebrafish (Beisaw et al., 2020), consistent with our findings. Currently, it is unclear how AP-1 is being recruited in Hdac7 OE CMs and whether this molecular switch from MEF2 to AP-1 is specific to certain cell types such as CMs; we will investigate these in future studies. It was quite intriguing that HDAC7 interacts with MCM complexes including MCM7 (Figure 5). MCM7 plays a critical role in DNA replication by unwinding DNA and increases its own expression by interacting with DNA-binding protein MCM1 at the replication origin, although MCM7 alone does not bind DNA (Fitch et al., 2003). This appears to be more plausible by acting on the very first step of the cell cycle to achieve full cell cycle activation. It is interesting to note that the interaction of HDAC7 with MCM complexes appears to be dependent on its interaction with MEF2. Again, this may suggest that dedifferentiation appears to be the prerequisite for CM proliferation.

Our findings of promotion of CM proliferation by HDAC7 support the idea that epigenetic manipulation may become a novel strategy to induce cardiac regeneration through transcriptional regulation (Cui et al., 2018; Soler-Botija et al., 2020; Kraus, 2022; Huang et al., 2023). On the other hand, cautions shall be exercised with respect to potential adverse off-target effects as epigenetic factors often have multiple downstream targets. Whether other class IIa HDAC members have similar effects on CM proliferation when they are overexpressed needs further investigation, although their expression persists in adult CMs (Zhang et al., 2002; Chang et al., 2004, 2006; Wright and Menick, 2016; Helmstadter et al., 2021; Han et al., 2022; Liu et al., 2022; Supplementary Figure S2).

We acknowledge that there are certain limitations in this current study. We have not tested *Hdac7* OE in adult heart *in vivo*. Considering that long-term *Hdac7* OE would induce excessive CM dedifferentiation and thus compromise CM contractility, utilizing transient adenoviral infection would be appropriate rather than a transgenic approach. However, there are also safety concerns on using adenoviral vectors to deliver genes of interest. For instance, adenoviral vectors can induce inflammatory responses, although many vaccines continue to utilize adenoviral vectors (Katz et al., 2016) and novel adenoviral vectors continue to be developed with the aim of improved efficacy and reduced side effects (Joshi et al., 2015). Other gene carriers such as nanoparticles merit consideration (Wei et al., 2024). In our current report, we primarily focused on testing the biological effects of Hdac7 OE on CMs via Ad5 adenoviral vector delivery and did not observe any apparent adenoviral vector-related adverse effects on CM function or structure (Supplementary Figure S2). In future studies, we will identify the optimal dose and time for *Hdac7* adenoviral treatment in injured adult hearts.

In summary, we show that HDAC7 promotes CM proliferation through a dual mechanism: dedifferentiation and cell cycle activation. HDAC7 accomplishes this dual action by redefining the transcriptional programs at the epigenetic/genetic level. Our findings shed new light on a novel approach to promote adult heart regeneration.

## Materials and methods

### Mice

CD1 mice were purchased from Charles River. All animal protocols were approved by the Nationwide Children's Hospital Research Institute Institutional Animal Care and Use Committee (IACUC, #AR22-00194).

### Adenovirus production

Adenovirus cloning and production of virus particles were conducted by using the pAdEasy Adenoviral Vector System (Luo et al., 2007). In our experiments, *Hdac7*-WT, *Hdac7*-dNLS, and *Hdac7*-L112A plasmids were obtained from Dr Hung-Ying Kao at Case Western Reserve University (Gao et al., 2010). *Hdac7* (wild-type or mutant) cDNAs were linked with mCherry fluorescence tag through P2A. The *Tnnt2* promoter (Addgene, #86558) and *Hdac7* cDNAs were cloned into pAdTrack (Addgene, #16404) and then underwent homologous recombination with pAdEasy-1 (Addgene, #16400) to generate recombinant viral DNAs. All recombinant adenoviral DNAs were digested with PacI and transformed into HEK293AD cells (Agilent Technologies, #240085) to produce viral particles using Lipofectamine 2000 (Invitrogen) and then purified and concentrated using the AdEasy Virus Purification Kit (Agilent) for further experiments. The titer is at 1 × 10^9^–5 × 10^9^ PFU/ml.

### NMCM culture and immunofluorescence staining

Primary mouse CMs were isolated from postnatal day 1 CD1 mice as previously described (Beall et al., 2023). Briefly, hearts were cut into small pieces, and ventricular tissues were digested overnight in trypsin solution (10 mM 4-(2-hydroxyethyl)piperazine-1-ethane-sulfonic acid [HEPES], 0.5 mM ethylenediaminetetraacetic acid [EDTA], 0.5% trypsin, and 1× Hank's balanced salt solution [HBSS]) at 4°C with shaking. Digestions were pooled, and cells were resuspended and centrifuged in dissociation buffer (10% (*w*/*v*) horse serum, 5% fetal bovine serum, 10 mM HEPES, and 1× HBSS). Cells were plated on tissue culture dishes and incubated for 1 h at 37°C in 5% CO_2_ to remove adherent cardiac fibroblasts. Supernatants containing CMs were replated on laminin-coated tissue culture dishes, incubated at 37°C in 5% CO_2_ for 2 days, and then transfected with recombinant adenovirus. After 3 days, cells were harvested for RNA extraction, protein extraction, or fixed with 4% paraformaldehyde for immunofluorescence staining. The percentages of Ki67^+^ or p-H3^+^ ACTC1^+^ NMCMs were quantified using Fiji software. Antibody information is listed in Supplementary Table S1.

### RNA isolation, qRT-PCR, and bulk RNA-seq

The RNeasy Plus Mini Kit (Qiagen) was used to extract total RNA. cDNA was generated using the Superscript III kit (Thermo Fisher Scientific). To detect mRNA, SYBR Green qRT-PCR was performed on a StepOnePlus Real-Time PCR System (Applied Biosystems) using PCR primers for genes of interest. qRT-PCR primers are listed in Supplementary Table S2. For bulk RNA-seq, samples were prepared following the provider's guidelines (Novogene) and sequenced on an Illumina NextSeq500. Sequencing reads were aligned to the UCSC mm10 reference genome using tophat2 and bowtie2 in R. Differential expression of transcripts was calculated using the cufflinks suite in R analyses.

### ATAC-seq and TOBIAS analysis

After 72 h of adenoviral transfection, NMCMs were washed once with cold phosphate-buffered saline and treated with DNase buffer for 30 min at 37°C prior to collection and cryopreservation at −80°C. Subsequent library preparation and sequencing were performed according to the manufacturer's protocol (Active Motif). Prediction of MEF2 and AP-1 binding was performed using the TOBIAS footprinting framework (https://github.com/loosolab/TOBIAS). Briefly, the ATAC-seq signals from single replicates were merged per condition and corrected for Tn5 bias using ‘TOBIAS ATACorrect’ with standard parameters. The corrected signals represent regions of more/less protection than would be expected by the background level of Tn5 insertions. Footprint scores and differential footprint scores (representing differential protein binding) between conditions were calculated using ‘TOBIAS ScoreBigwig’ and ‘TOBIAS BINDetect’ across a merged set of all genomic regions with open chromatin as described (Bentsen et al., 2020).

### Immunoblotting

NMCMs were collected in cold lysis buffer containing 50 mM Tris–HCl, pH 7.4, 150 mM NaCl, 1 mM Na_2_EDTA, 1 mM ethylene glycol tetraacetic acid, 1% Triton X-100, 0.5% sodium deoxycholate, 0.1% sodium dodecyl sulphate (SDS), and protease and phosphatase inhibitor cocktail (Roche), and 1 mM phenylmethylsulfonyl fluoride was added before use. Protein samples were resolved on 4%–12% SDS–polyacrylamide gel electrophoresis (SDS–PAGE) before transferring to polyvinylidene fluoride membranes. Membranes were incubated with primary antibodies at 4°C overnight. Detailed information of antibodies is listed in Supplementary Table S1. Target proteins were visualized by chemiluminescence using horseradish peroxidase-conjugated secondary antibodies. The density of protein bands was quantified by ImageJ software.

### Co-IP

NMCMs were transfected with *Hdac7*-WT, *Hdac7*-dNLS, or *Hdac7*-L112A adenoviruses for 12 h, and then washed and cultured for another 36 h with regular media. Cells were lysed at 4°C in Pierce IP Lysis Buffer (Thermo Fisher Scientific) supplemented with protease inhibitor cocktail (Roche). Whole lysate (1 mg) was subjected to the standard co-IP procedure. Briefly, magnetic beads were pre-cleared with normal IgG and then incubated with mouse-FLAG antibody for 6 h at 4°C. Lysates were precipitated with antibody-conjugated beads by overnight incubation. Precipitated proteins were resolved by SDS–PAGE and subsequently immunoblotted with HDAC7, MEF2D, MCM3, MCM5, and MCM7 antibodies, or MS analysis was performed.

### Proteomics sample preparation and LC–MS analysis

Protein lysates from NMCMs were immunoprecipitated by FLAG antibody. Beads containing affinity-purified proteins were resuspended with 8 M urea, 50 mM ammonium bicarbonate. Protein disulfide bonds were reduced with 5 mM tris(2-carboxyethyl) phosphine at 30°C for 60 min, and cysteines were subsequently alkylated with 15 mM iodoacetamide in the dark at room temperature for 30 min. Urea was then diluted to 1 M urea using 50 mM ammonium bicarbonate, and proteins were subjected to overnight digestion with MS-grade Trypsin/Lys-C mix (Promega). Following overnight digestion, beads were pulled down and the solution with peptides transferred to a new tube. Finally, samples were acidified with formic acid (FA) and subsequently desalted using AssayMap C18 cartridges mounted on an Agilent AssayMap BRAVO liquid handling system. Cartridges were first conditioned with 100% acetonitrile (ACN) followed by 0.1% FA. Samples were then loaded, washed with 0.1% FA, and eluted with 60% ACN, 0.1% FA. Finally, the organic solvent was diluted to allow peptide quantification using a NanoDrop spectrophotometer (Thermo Fisher Scientific), and then the remaining of the sample was dried in a SpeedVac concentrator. Prior to LC–MS/MS analysis, dried biotin-enriched peptides were reconstituted with 2% ACN, 0.1% FA, and concentration was determined using a NanoDrop spectrophotometer. Samples were then analyzed by LC–MS/MS using a Proxeon EASY-nLC system (Thermo Fisher Scientific) coupled to a Q-Exactive Plus Mass Spectrometer (Thermo Fisher Scientific). Peptides were separated using an analytical C18 Aurora column (75 μm × 250 mm, 1.6 μm particles; IonOpticks) at a flow rate of 300 nl/min (60°C) using a 120-min gradient: 1% to 5% B in 1 min, 6% to 23% B in 72 min, 23% to 34% B in 45 min, and 34% to 48% B in 2 min (A = 0.1% FA; B = 80% ACN:0.1% FA). The mass spectrometer was operated in positive data-dependent acquisition mode. MS1 spectra were measured in the Orbitrap in the mass-to-charge (*m/z*) of 350–1700 with a resolution of 70000 at *m*/*z* 400. The automatic gain control (AGC) target was set to 1 × 10^6^ with a maximum injection time of 100 ms. Up to 12 MS2 spectra per duty cycle were triggered, fragmented by high-energy collisional dissociation, and acquired with a resolution of 17500 and an AGC target of 5 × 10^4^, an isolation window of 1.6 *m*/*z* and a normalized collision energy of 25. The dynamic exclusion was set to 20 sec with 10 ppm mass tolerance around the precursor. All mass spectra were analyzed with MaxQuant software version 1.6.11.0. MS/MS spectra were searched against the Mus musculus UniProt protein sequence database (downloaded in January 2020) and GPM cRAP sequences (commonly known protein contaminants). Precursor mass tolerance was set to 20 ppm and 4.5 ppm for the first search where initial mass recalibration was completed and for the main search, respectively. Product ions were searched with a mass tolerance 0.5 Da. The maximum precursor ion charge state used for searching was 7. Carbamidomethylation of cysteine was searched as a fixed modification, while oxidation of methionine and acetylation of protein N-terminal were searched as variable modifications. Enzyme was set to trypsin in a specific mode and a maximum of two missed cleavages was allowed for searching. The target-decoy-based false discovery rate (FDR) filter for spectrum and protein identification was set to 1%. Relative protein abundances were calculated by the two normalization strategies available in MaxQuant: MaxLFQ29 and intensity-based absolute quantification (iBAQ). The values obtained by label-free quantification (LFQ) and iBAQ were highly consistent. The LFQ values were chosen for subsequent analyses.

### Quantification and statistical analyses

All experiments were independently repeated at least three times, and the number of samples (*n*) is stated in figure legends. Results are reported as the mean ± standard deviation. For statistical analyses, normal distribution of data was assessed by Shapiro–Wilk's test (when *n* ≥ 6). For data with small sample size (*n* < 6), non-parametric tests were applied. For parametric data with normal distribution, the statistical significance of the difference was assessed using unpaired two-tailed Student's *t*-test between two groups and the Tukey or Dunnett *post hoc* test for one-way analysis of variance (ANOVA). For non-parametric data or data with small sample size (*n* < 6), the Mann–Whitney *U* test and Kruskal–Wallis test for one-way ANOVA followed by the Dunn post *hoc test* were performed for comparisons between two groups. Specific statistical tests are stated in figure legends. GO enrichment analyses were performed using the Database for Annotation, Visualization and GOATOOLS bioinformatics resources (Klopfenstein et al., 2018). Relative protein expression on western blot data was quantified by densitometry. Fold changes for relative gene or protein expression were derived through division of values in the experimental group by the mean values from the control group. A *P*-value <0.05 was considered statistically significant. All statistical analysis was performed using GraphPad Prism 8 (GraphPad Software). Images with the value closest to the mean value were selected as representative.

## Supplementary Material

mjae044_Supplemental_File

## Notes

**Author contributions:** J.J. and D.L. conceived and designed the study, analyzed the experiments, and wrote the paper. M.B. and M.L. performed bulk RNA-seq and ATAC-seq data analysis. J.J, J.B., and L.C. performed the experiments. A.R.C. performed proteomics data analysis. J.J. and D.L. edited the manuscript. All authors reviewed the results and approved the final version of the manuscript.
