# Supplementary material for: HDAC7 promotes cardiomyocyte proliferation by suppressing myocyte enhancer factor 2
Source: J Mol Cell Biol. 2024 Oct 11;16(10):mjae044. doi: 10.1093/jmcb/mjae044 (PMC12059635; doi:10.1093/jmcb/mjae044)
Supplement: mjae044_Supplemental_File [file mjae044_supplemental_file.pdf]

Supplemental Figures and Figure Legends

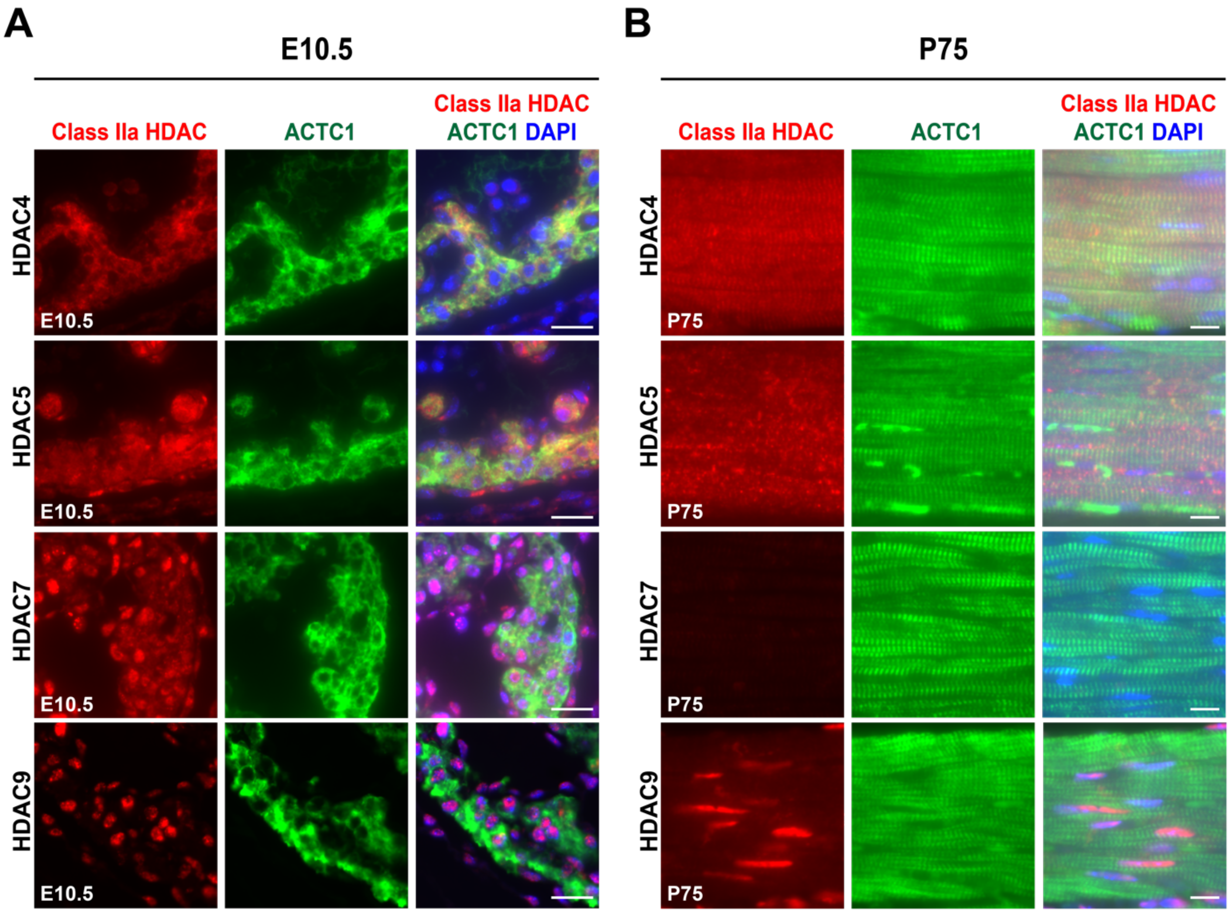

Supplemental Figure 1. Expression of Class IIa HDACs in embryonic and adult mouse hearts detected by immunofluorescence staining.

A. E10.5 embryonic hearts. Scale bars, 25 μm. **B.** P75 adult hearts. Scale bars, 10 μm.

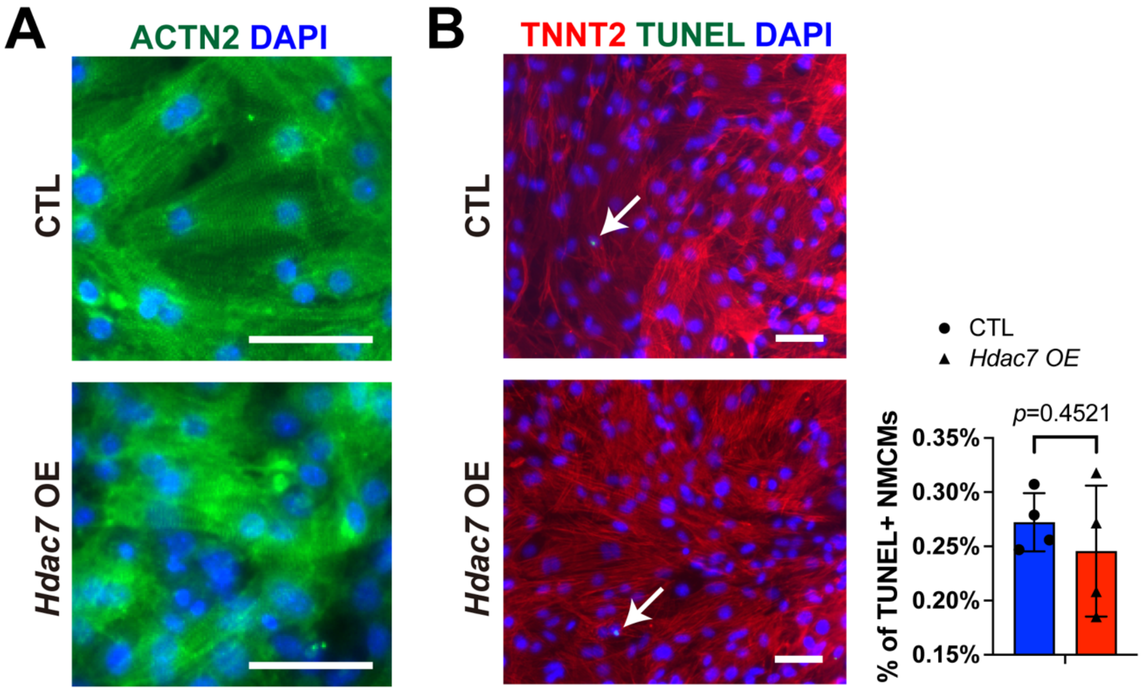

Supplemental Figure 2. Normal sarcomere and no excessive cell death in *Hdac7* overexpressed neonatal mouse cardiomyocytes (NMCMs) cultured for 7 days.

A. Immunofluorescence staining of α-ACTININ-2 (ACTN2, a sarcomere protein) of cultured NMCMs 7-days post-transfection of either adenoviral *Hdac7* (Hdac7 OE) or mCherry control (CTL) adenoviruses. Scale bars, 50 μm. **B.** Co-immunostaining of TNNT2 and TUNEL of cultured NMCMs 7-days post-transfection of either *Hdac7* or mCherry adenoviruses. Arrows point to the TUNEL+ NMCMs. Scale bars, 50 μm. Quantification of percentage of TUNEL+ NMCMs is shown on the right. *P*-values were determined by unpaired Student's t-test.

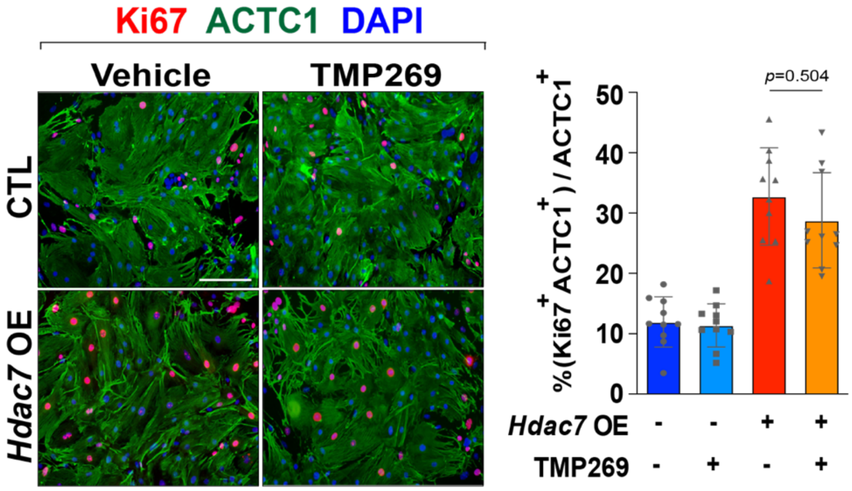

Supplemental Figure 3. Deacetylase activity of HDAC7 is dispensable for Hdac7 to induce cardiomyocyte proliferation.

Neonatal mouse cardiomyocytes (NMCMs) were treated with either *Hdac7* overexpression (*Hdac7* OE) or mCherry (CTL) adenovirus (working titer: $1X10^7$ GC/ml) in the presence or absence of TMP269 (Cat. #1314890-29-3, Cayman chemical, a specific HDAC7 inhibitor, working concentration: 36 nM) for 3 days. Cell proliferation was assessed by the percentage of Ki67+ NMCMs. Scale Bar, 125 µm. Quantification is shown on the right. n=10 in each group. *P*-values were determined by One-way ANOVA followed by Tukey post hoc test.

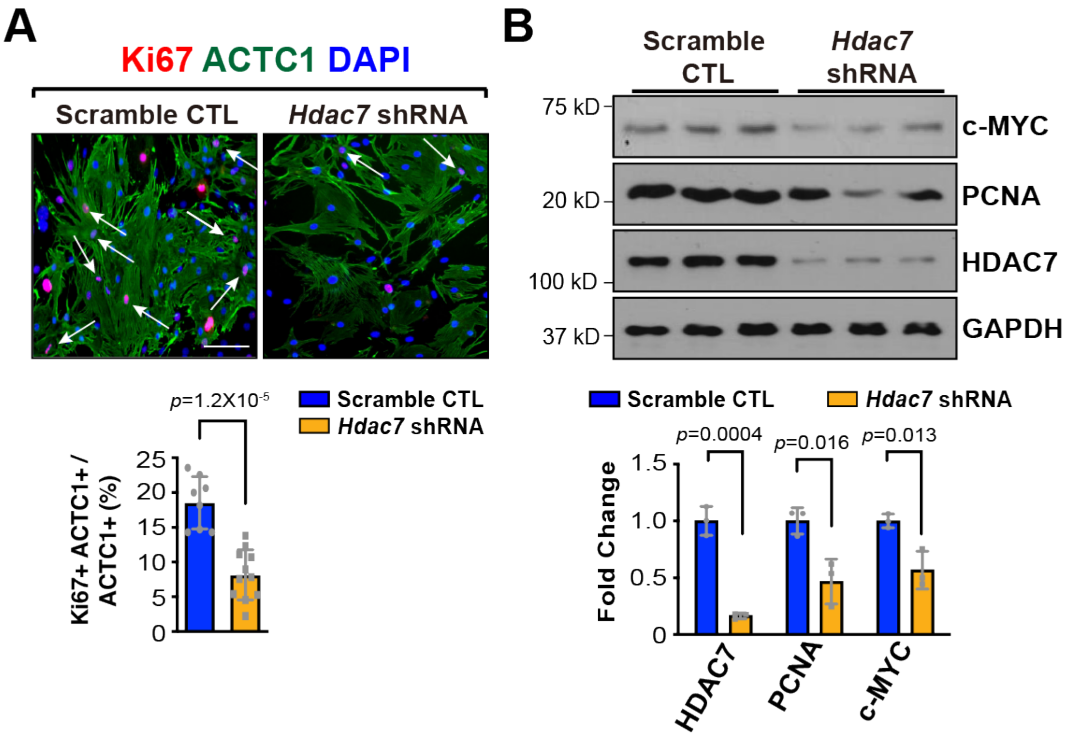

Supplemental Figure 4. Knockdown of HDAC7 in NMCMs resulted in decreased CM proliferation.

A. CM proliferation (percentage of Ki67+ CMs) was significantly decreased when *Hdac7* was knocked down by shRNA for 3 days. Scale bar: 125 µm. n=8 for scramble control (CTL), n=11 for *Hdac7* shRNA. *P*-values were determined by unpaired Student's t-test. **B.** c-MYC and PCNA were significantly decreased in *Hdac7* knockdown NMCMs determined by western blotting. n=3 for each group. *P*-values were determined by unpaired Student's t-test.

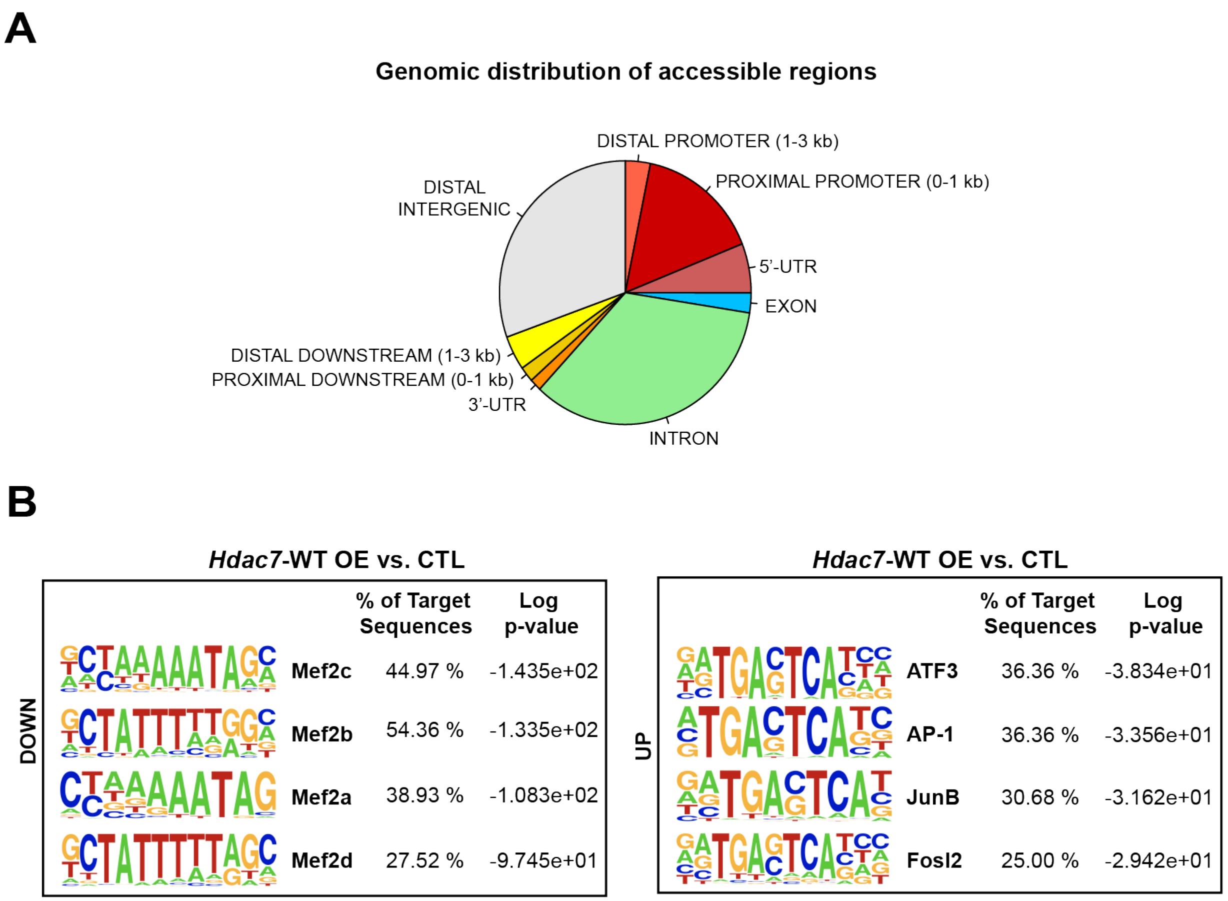

Supplemental Figure 5. ATAC-seq analysis in CTL and HDAC7 overexpressed cardiomyocytes.

A. Genomic distribution of ATAC-seq in *Hdac7*-WT overexpressed (*Hdac7*-WT OE) neonatal mouse cardiomyocytes (NMCMs). **B.** Analysis of homer motif enrichment analysis in genomic regions with increased accessibility in *Hdac7*-WT OE NMCMs.

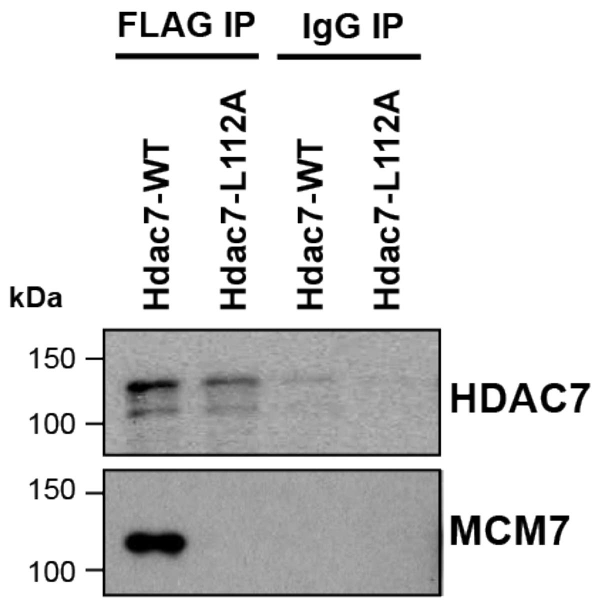

Supplemental Figure 6. ***Hdac7*****-L112A does not interact with MCM7.**

Co-immunoprecipitation (Co-IP) of MCM7 using Flag antibody. Cell protein lysates were collected 3 days post-transfection of NMCMs by *Hdac7*-WT (positive control), *Hdac7*-112A. 500 µg of total protein was used for Co-IP.

Supplemental Table 1. Antibodies

Target antigen	Vendor or Source	Catalog #	Working concentration	Lot # (preferred but not required)	Persistent ID / URL
**FLAG**	Sigma	F1804 1MG	IHC-1:50<br>WB-1:5000<br>IP: 1:500	124K6106	https://www.sigmaaldrich.com/US/en/product/sigma/f1804
**Ki67**	Abcam	ab15580	IHC-1:200<br>WB-1:1000	GR53793-1	https://www.abcam.com/products/primary-antibodies/ki67-antibody-ab15580.html
**mCherry**	Novus	NBP2-25157	IHC-1:100		https://www.novusbio.com/products/mcherry-antibody_nbp2-25157
**ACTC1**	Abcam	Ab46805	IHC-1:400<br>WB-1:1000	GR106692-3	https://www.abcam.com/muscle-actin-antibody-ep184e-ab46805.html
**PCNA**	Fisher Thermo Scientific	13-3900	IHC-1:100<br>WB-1:1000		https://www.thermofisher.com/antibody/product/PCNA-Antibody-clone-PC10-Monoclonal/13-3900
**p-H3**	Cell Signaling	9701s	IHC-1:100		https://www.cellsignal.com/products/primary-antibodies/phospho-histone-h3-ser10-antibody/9701
**AURKB**	Abcam	Ab2254	IHC-1:100<br>WB-1:1000	GR129671-1	https://www.abcam.com/aurora-b-antibody-ab2254.html
**ACTN2**	Proteintech	14221-1-AP	IHC-1:200<br>WB-1:1000		https://www.ptglab.com/products/ACTN2-Antibody-14221-1-AP.htm
**TNNT2**	Thermo Scientific	Ms-295-P1	IHC-1:200<br>WB-1:1000	295P1510F,<br>295P1608H	https://www.thermofisher.com/order/catalog/product/MS-295-P
**GAPDH-HRP**	Proteintech	HRP-60004	WB-1:10,000		https://www.ptglab.com/products/GAPDH-Antibody-HRP-60004.htm
**MCM3**	Cell Signaling	4012S	WB-1:1000	2	https://www.cellsignal.com/products/primary-antibodies/mcm3-antibody/4012
**MCM5**	Proteintech	11703-1-AP	WB-1:1000		https://www.ptglab.com/products/MCM5-Antibody-11703-1-AP.htm
**MCM7**	Cell Signaling	3735S	WB-1:1000	3	https://www.cellsignal.com/products/primary-antibodies/mcm7-d10a11-xp-rabbit-mab/3735
**HDAC4**	Thermo Scientific	PA5-29103	IHC-1:200	WA3164769B	https://www.thermofisher.com/antibody/product/HDAC4-Antibody-Polyclonal/PA5-29103
**HDAC5**	Proteintech	16166-1-AP	IHC-1:100	00019488	https://www.ptglab.com/products/HDAC5-specific-Antibody-16166-1-AP.htm
**HDAC7**	Invitrogen	PA5-104214	IHC-1:400	WD3265244A	https://www.thermofisher.com/antibody/product/PA5-104214.html?CID=AFLAP-PA5-104214
**HDAC9**	Invitrogen	PA5-78197	IHC-1:100	XA3488340C	https://www.bio-techne.com/p/antibodies/tgf-beta3-antibody-20724_mab243#technical-data-tab-tab
**Mouse-IgG**	Abcam	Ab170326	IP-1:500		https://www.abcam.com/hrp-kappa-light-chain-antibody-kt102-ab170326.html
**Mouse-HRP**	Cell Signaling	7076	WB-1:5000		https://www.cellsignal.com/products/secondary-antibodies/anti-mouse-igg-hrp-linked-antibody/7076
**Rabbit-HRP**	Cell Signaling	7074	WB-1:5000		https://www.cellsignal.com/products/secondary-antibodies/anti-rabbit-igg-hrp-linked-antibody/7074?site-search-type=Products&N=4294956287&Ntt=7074s&fromPage=plp&_requestid=2127397

Supplemental Table 2. qRT-PCR Primers

Description	Forward	Reverse
mHdac7	GCAGCCCTTGAGAGAACAGT	TGTCCAAGGGCTCAAGAGTT
mPcna	CTAGCCATGGGCGTGAAC	GAATACTAGTGCTAAGGTGTCTGCAT
mKi67	AGGGTAACTCGTGGAACCAA	TCTTAACTTCTTGGTGCATACAATG
mAurkb	TCGCTGTTGTTTCCCTCTCT	TTCAGGCCAGACTGAGACG
mMyc	CCTAGTGCTGCATGAGGAGA	TCCACAGACACCACATCAATTT
mPlk1	TTGTATTTTTGGAGCTCTGTCG	CCTGCAGTACATAGAGCGTGA
mCycb1	TGCATTTTGCTCCTTCTCAA	CAGGAAGCAGGGAGTCTTCA
mCycd1	CCTTTGTGGCCCTCTGTG	CAGGTTCAGGCCTTGCAT
mCycd2	GGCCAAGATCACCCACACT	ATGCTGCTCTTGACGGAACT
mE2f1	ATCCCAGTCAATCCCTGTTG	TGGTGACAGTTGGTCCTCTTC
mActn2	AGCAGCAGAGGAAGACGTTC	ATGTTCTCGATCTGGGTGCC
mTnni3	ATGACCTCCGTGGCAAGTTT	GCCCTCAGGTCCAAGGATTC
mActc1	TGAAGCCTCACTTCCTACCC	GCTTTGGTGGGTTCTGTAGG
mNppa	CACAGATCTGATGGATTTCAAGA	CCTCATCTTCTACCGGCATC
mNppb	GTCAGTCGTTTGGGCTGTAAC	GGAAAGAGACCCAGGCAGA
mMcm3	TGTGGAGATGAGGCGAGAG	TGTCTACCATCCGAAGTCGAG
mMcm5	CTTTGTCTGGCAATCTGTCG	AAATGCGGCTCAGCATCTC
mMcm7	CCCTGCCCAATTTGAACCTTT GGA	TCT CCA CAT ATG CTG CGG TGA TGT
mGAPDH	TCCTGGTATGACAATGAATACGGC	TCTTGCTCAGTGTCCTTGCTGG
